# The spread of Injectate after ultrasound-guided lateral elbow injection – a cadaveric study

**DOI:** 10.1186/s40634-018-0142-8

**Published:** 2018-07-18

**Authors:** Jonathan P. Evans, Jeremy Metz, Rahul Anaspure, William J. Thomas, Andrew King, Vicki A. Goodwin, Chris D. Smith

**Affiliations:** 1 Royal Devon and Exeter NHS Foundation Trust, Exeter, UK; 2 Wellcome Trust Biomedical Informatics Hub, University of Exeter, Exeter, UK; 3 National Institute for Health Research (NIHR) Collaboration for Leadership in Applied Health Research and Care (CLAHRC) South West Peninsula, University of Exeter Medical School, Exeter, UK; 4 Health Services and Policy Research, Smeall Building, JS03, St Lukes Campus, Exeter, EX1 2LU UK

## Abstract

**Background:**

Injections into the tendinous portion of the common extensor origin are a common intervention in the treatment of Lateral Elbow Tendinopathy (LET). Clinical trials report a heterogeneous selection of injectate volumes and delivery techniques, with systematic reviews finding no clear consensus. The aim of this study was to assess the intratendinous distribution and surrounding tissue contamination of ultrasound-guided injections into the Common Extensor Tendon (CET) of the elbow.

**Methods:**

Twenty cadaveric elbows were injected by a Consultant Radiologist under Ultrasound guidance. Elbows were randomised to equal groups of 1 or 3 mls of methylene blue injection, delivered using single shot or fenestrated techniques. Following injection, each cadaver underwent a dry arthroscopy and dissection of superficial tissues. The CET was excised, set and divided into 1 mm sections using microtome. Each slice was photographed and analysed to assess spread and pixel density of injectate in four colour graduations. The cross-sectional area of distribution was calculated and compared between groups.

**Results:**

In all 20 cadaveric samples, contamination of the joint was noted on arthroscopy and dissection. Injectate spread through over 97% of the cross-sectional area. No differences were found in intratendinous spread of injectate between differing volumes or techniques.

**Conclusion:**

This study found that commonly used injection volumes and techniques distribute widely throughout cadaveric CETs. There was no improvement when the volume was increased from 1 to 3 mls or between single shot of fenestrated injection techniques. It should be noted that joint contamination using these techniques and volumes may be inevitable.

**Electronic supplementary material:**

The online version of this article (10.1186/s40634-018-0142-8) contains supplementary material, which is available to authorized users.

## Background

Injection therapy for chronic lateral elbow tendinopathy (LET), known commonly as tennis elbow, remains a popular treatment choice (Sahbudin & Peall, 2013; Titchener et al., 2015). Though corticosteroids were historically the most common preparation, recent evidence of its negative long-term sequelae (Bisset et al., 2011) may see its usage decline. However, the emergence of novel therapies such as platelet-rich plasma (PRP), autologous blood (AB), botulinum toxin, glycosaminoglycan polysulphate, sodium hyaluronic or prolotherapy continue to promote interest in injection treatment. Systematic reviews of these therapeutic options remain inconclusive, with a recurring criticism of the heterogeneity of injection dosing and technique between studies (Ahmad et al., 2013; de Vos et al., 2014; Dong et al., 2016; Fitzpatrick et al., 2017; Long et al., 2015).

Pathological change in LET occurs within the proximal tendons of the common wrist extensor muscles, with particular reference to the extensor carpi radialis brevis (ECRB). Hence this is the intended site of injection therapy in LET. The ECRB tendon originates from the lateral epicondyle, lying deep to the remaining common extensor tendons and superficial to the thin articular capsule of the elbow (Nimura et al., 2014). Injection volumes delivered to this area commonly range from 0.5–3.5mls (Dong et al., 2016) and employ either a single shot or fenestrated (pepper pot) administration techniques (Dong et al., 2016; Fitzpatrick et al., 2017). Cadaveric assessment has only been undertaken for anatomically guided injections (Keijsers et al., 2017). The injections were delivered by experienced clinicians using their standard techniques, the study reported poor localisation of injectate, within only 33% (partially) localised to the ECRB tendon and 60% localised intra-articular.

The location of the injectate in lateral elbow injections is of clear importance. Under the premise that many of these substances confer benefit due to their active constituents, it is of vital importance that the retention and distribution of injectate within the tendon is quantified, and furthermore that the commonly employed volumes and techniques are compared. Assessment of the contamination of joint space and surrounding tissues is also warranted owing to the potentially noxious or unwanted effects of botulinum toxin, or the potential chondrolytic effects of corticosteroid (McAlindon et al., 2017) and local anaesthetics (Piper et al., 2011). This study aims to determine the intratendinous distribution and surrounding contamination of commonly utilised injection volumes and techniques, delivered under ultrasound guidance, in cadaveric specimens. It was hypothesised that ultrasound guidance would ensure accurate delivery to the common extensor tendons and that intratendinous distribution was dose-dependent and improved with fenestrated techniques.

## Methods

In this cadaveric study, 20 fresh-frozen, unembalmed upper-limb specimens from 10 individuals were used. Age of the specimens ranged from 70 to 96 years, four were female, and six were male. The specimens were sectioned at the upper 3rd of the humerus proximally and radio-carpal joint distally. Specimens had not undergone previous upper limb surgery. Information regarding any history of tendinopathy or other pathological abnormalities was not known by the authors. Ethical approval (REC 17/NW/0065) was obtained from the NHS North West - Preston Research Ethics Committee.

### Injections technique

**Fig. 1:**
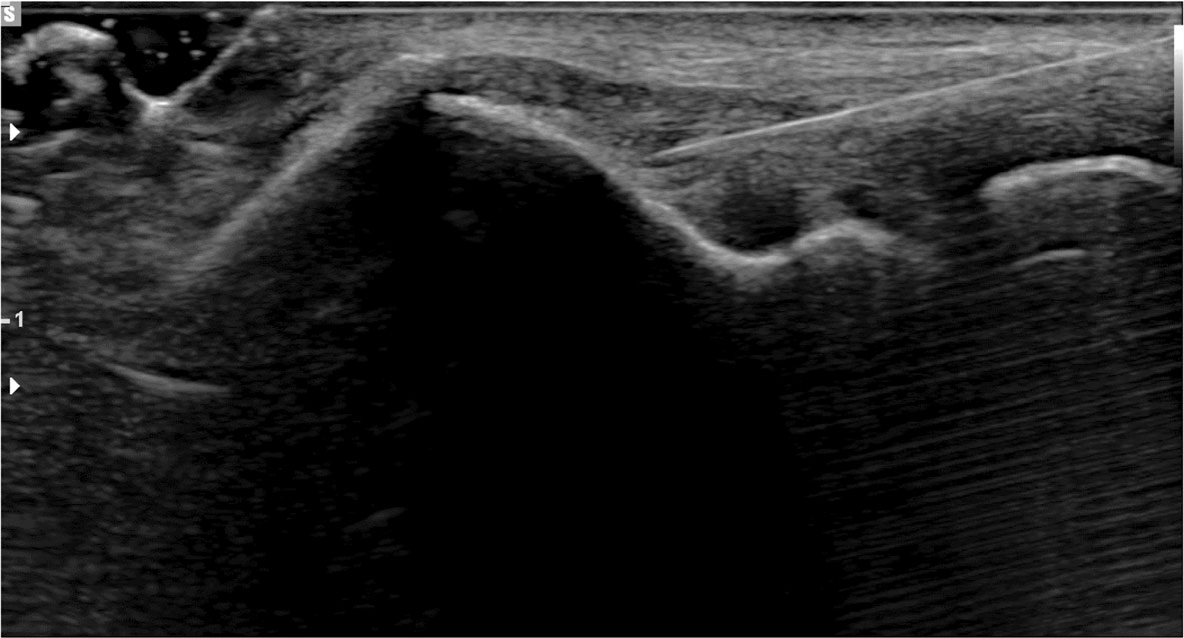
Longitudinal ultrasonogram of the Common Extensor Tendon (CET). The hypodermic needle can been seen entering at the right side of the image. This particular specimin underwent a 1 ml single shot injection

The specimens were block randomised to receive either a 1 or 3 ml (ml) injection, delivered using a single pass or fenestrated technique, yielding four groups of five specimens. Injection volume was derived from the 25% and 75% percentile of injection volumes from studies reported in a recent comprehensive systematic review (Dong et al., 2016). The single pass technique delivered the injection into the mid portion of the of the anterior Common Extensor Tendon (CET) origin, corresponding to the position of the ECRB (Konin et al., 2013) and the most commonly injected position, the fenestrated technique used nine passes delivered in a 3 × 3 square pattern across the anterior CET origin. The injected material was 2.44% methylene blue; all injections were delivered using a 5 ml syringe and 21 gauge needle. Injections were delivered by a Consultant musculoskeletal radiologist, with 6 years’ experience, using a Siemens RS80A ultrasound machine (Seimens, Munich, Germany) using a16 MHz transducer in both transverse and longitudinal planes (Fig. 1). The prosections were positioned with the elbow flexed to 45–50°. Evidence and size of intrasubstance and footprint tendon tears before and after injection and calcification was quantified using ultrasound.

### Anatomic dissection

Following injection the elbows were positioned with the arm over a bar, mimicking the lateral decubitus position. The specimen was held with a single clamp on the skin overlying the triceps muscle. Dissection was preceded by a dry arthroscopy using a single high proximal anteromedial portal. The presence of intra-articular joint contamination of injectate was recorded.

**Fig. 2:**
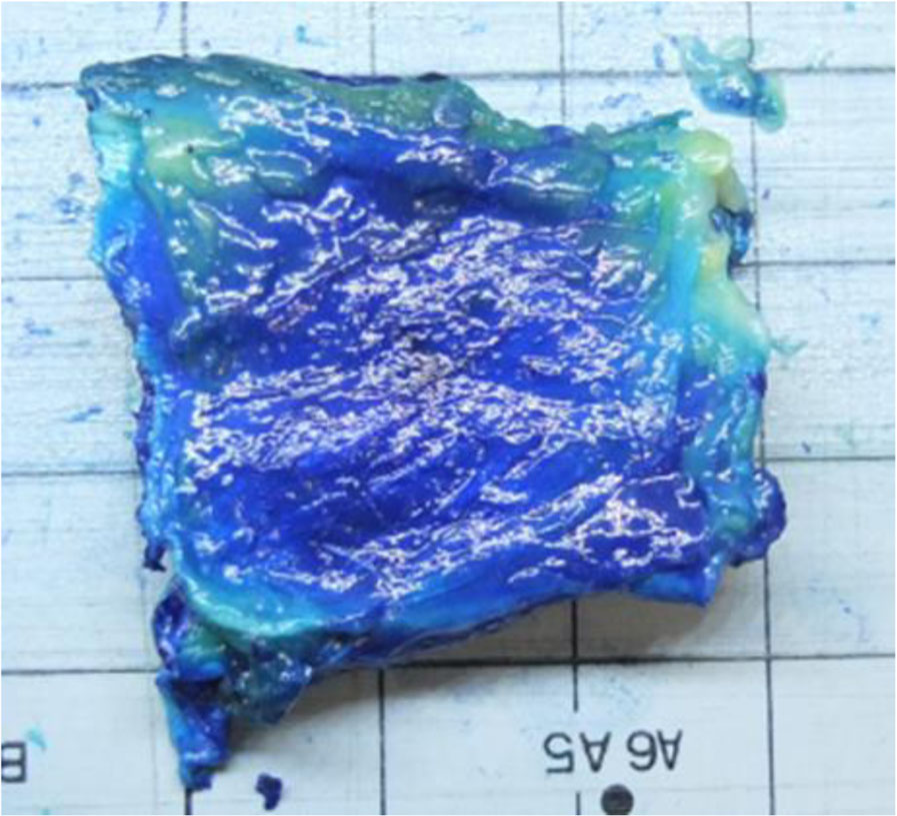
Dissected Common Extensor Tendon (CET). Showing non-articular side. Blue colouration from methylene blue dye injection

Dissection was performed through a posterior midline incision, with subdermal excursion to the lateral side. Soft tissue contamination was recorded. The CET was identified. This was excised proximally subperiosteally to the lateral epicondyle, and distally at least 1 cm distal to the musculotendinous interface. The excised CET was transferred to a dissection table where periosteal tissue was removed from the insertion, the musculotendinous junction was identified and tissue distal to it removed, leaving the isolated CET (Fig. 2). The macroscopic appearance of the CET was digitally photographed and recorded.

### Image analysis

The dissected CETs were placed in microtome template and surrounded with low melting point paraffin wax. Specimens were stored in the dissection room cold store at 6 °C for 12 h. The specimens were then mounted in a bench microtome (Brunel Microscopes Ltd., Chippenham, UK) and were sectioned in an axial plane at 1-mm intervals. Each section was digitally photographed using a static high-resolution 12-megapixel camera (Fujifilm, Tokyo, Japan).

**Fig. 3:**
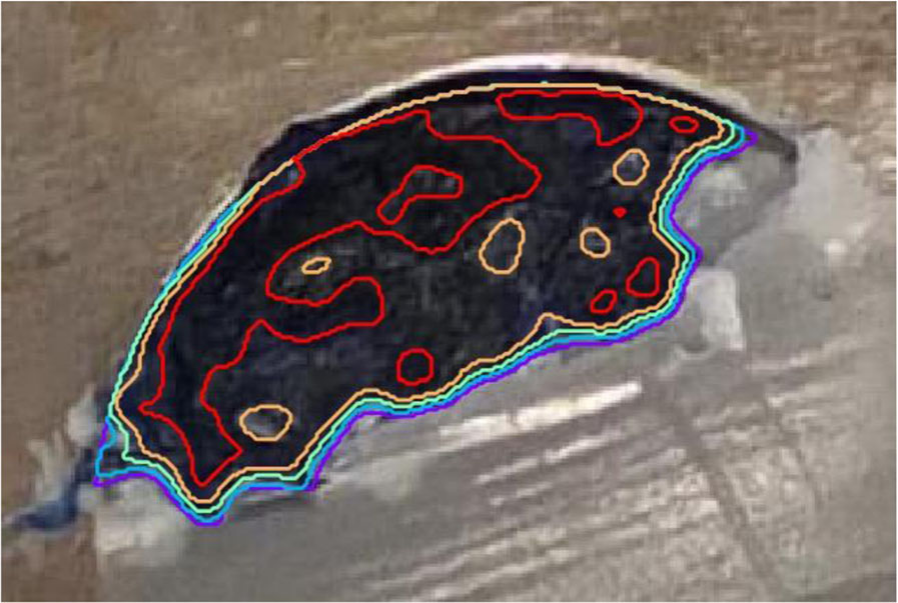
Common Extensor Tendon (CET) held in wax surround of bench microtome. The four areas of algorithmically derived colour intensity are seen within each of the four contour lines

Each digital photograph underwent a two-stage image analysis process. Semi-automated segmentation of the tendon border from the wax surround was undertaken and lighting normalised between sections using the median intensity of the region outside the tendon/wax specimen. Following this, algorithmic contour lines denoting intensity of the dye (i.e. the retention of the methylene blue from light blue (distributed dye) to dark blue (concentrated dye) in four increments representing quartiles of the blue colour spectrum) were overlaid using the red channel, following light smoothing with a Gaussian filter (Fig. 3). The second stage quantified the total number of pixels denoting the tendon area, and subsequently, the number of pixels within the four increments of dye intensity was quantified. Pixel number was transformed to fractional area to allow comparison within and between the tendon samples. Finally, the slices were reformatted using the marching cubes algorithm to provide 3-dimensional representations which were visually assessed for patterns of injectate spread and pooling.

### Statistical analysis

The primary outcome, percentage of pixels, was reported descriptively using means and standard deviation. Further analysis was performed using hierarchical linear regression modelling, with a fixed effect on injection technique, injection volume, and dye intensity, and a random effect on the cadaveric specimen, nested within patients. Each specimen was tested under each of the two conditions (technique and volume) with pixel percentage reported in four gradations (density of blue dye). Regression coefficients and 95% Confidence Intervals (CI) are reported and statistically significant differences in pixel percentage between injection technique and volume was defined as a global *p* < 0.05 for catagorical variables. All statistical analysis was undertaken using Stata 14 (StataCorp. 2015. Stata Statistical Software: Release 14. College Station, TX: StataCorp LP).

## Results

Pre-injection ultrasound identified that 60% of the 20 cadavers had CET tears, 33% of those tears were located at the footprint and an average size on ultrasound measurement of 5.8 mm (Range 6-8 mm). Of the eight CETs without a pre-injection tear, the post-injection ultrasound identified a tear in five (62.5%). Intra-tendinous calcification was evident in seven (53.8%) of the CETs. Following injection, intra-articular elbow joint contamination was evident in all 20 specimens on dry arthroscopy. Macroscopic assessment consequently found contamination in both the lateral and medial joint space and on the articular and non-articular sides of the CET.

The appearance of the external surface of all 20 CETs demonstrated the focus of the dye at the tendon site with widespread surrounding soft tissue contamination that diminished in proportion to the distance from the injection position.

The mean volume (mm^3^) of the CET specimens, derived from the total pixel density, was 1040mm^3^ (±371.91 Range 344.72mm^3^ to 1845.74mm^3^). When separated by group (injectate volume or technique), no statistical differences in tendon volume were found.

**Fig. 4:**
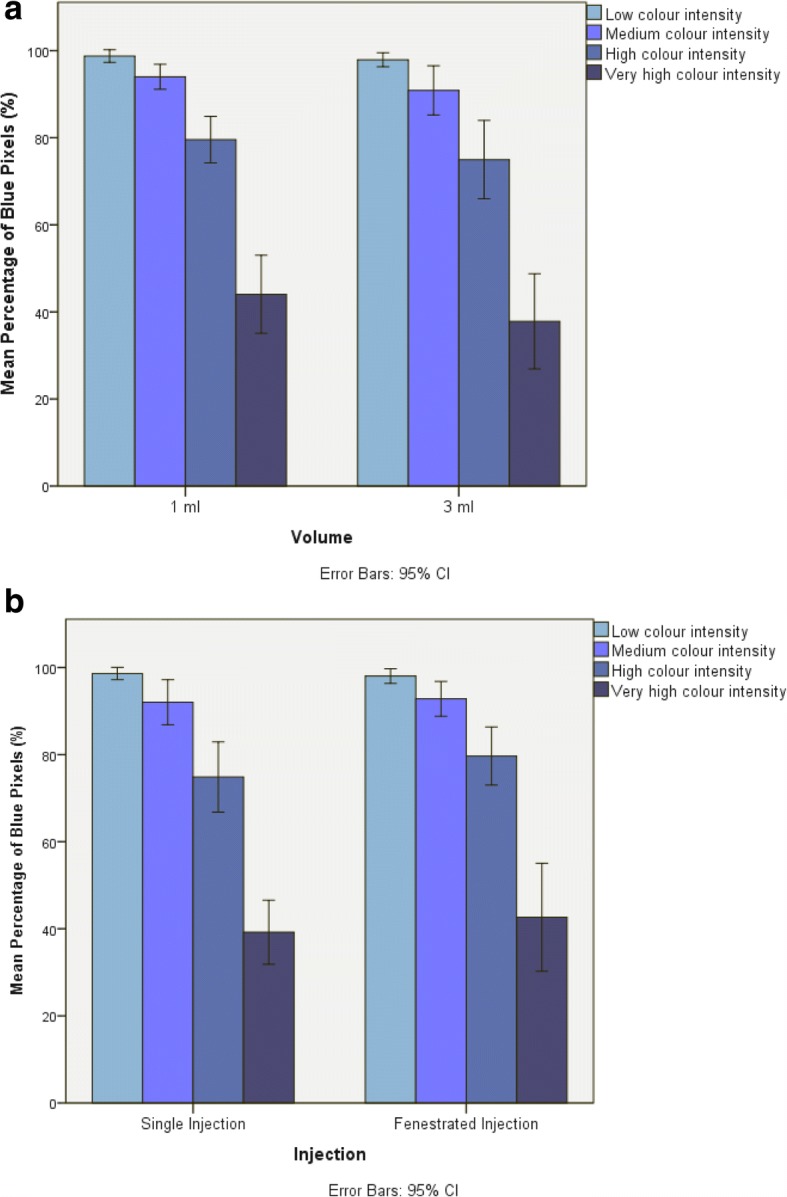
**a** Bar chart showing the mean percentage of blue pixels in each of the four colour intensity groups for the 1 ml and 3 ml volume injections. Error Bars = 95% Confidence Intervals. **b** Bar chart showing the mean percentage of blue pixels in each of the four colour intensity groups for the single injection and fenestrated injection techniques. Error Bars = 95% Confidence Intervals

The mean percentage of intratendinous pixel density, at the most sensitive dye intensity (lightest blue), was 98.76% (±2.0) for 1 ml and 97.91% (±2.27) for 3 ml, 98.63% (±1.96) for single shot injections and 98.05% (±2.35) for fenestrated injection. Mean percentage of blue dye concentration, in the four colour intensities from lightest blue to darkest blue is shown in Table 1 and graphically in Fig. 4. Statistically significant differences were found between the groups of blue pixel distribution against the baseline of group 1 (group 2 regression coefficient − 0.06 (95% CI -0.10 to − 0.02), group 3–0.21 (− 0.25 to − 0.17) and group 4–0.60 (− 0.61 to − 0.54)) with a global *p*-value of < 0.001. However, no statistically significant differences in blue pixel distribution were found between for the dependent variables injection volume (*p* = 0.255, 95% CI -0.10 to 0.03) or injection technique (*p* = 0.514 95% CI -0.04 to 0.08). Potential differential effects of brightness level for different injection types and volumes were investigated by addition of an interaction term between brightness and injection type/volume (only one interaction term was included per model). No differential effects of brightness across injection type/volume were observed.

**Table 1:** Mean percentage +/− Standard deviation (SD) of the Blue pixels distributed within the CET tendon from lightest blue to darkest blue in four groups of blue colour graduation (lightest blue = group 1, darkest blue = group 4))

Injection Group	Blue pixel distribution							
	Lightest blue ←→ Darkest blue							
	Group 1		Group 2		Group 3		Group 4	
	Mean	SD	Mean	SD	Mean	SD	Mean	SD
1 ml	98.76%	2.00%	94.00%	4.01%	79.55%	7.49%	44.04%	12.54%
3 ml	97.91%	2.27%	90.87%	7.92%	74.98%	12.59%	37.83%	15.30%
Single shot	98.63%	1.96%	92.04%	7.26%	74.86%	11.25%	39.21%	10.29%
Fenestrated	98.05%	2.35%	92.82%	5.59%	79.67%	9.32%	42.66%	17.33%

**Fig. 5:**
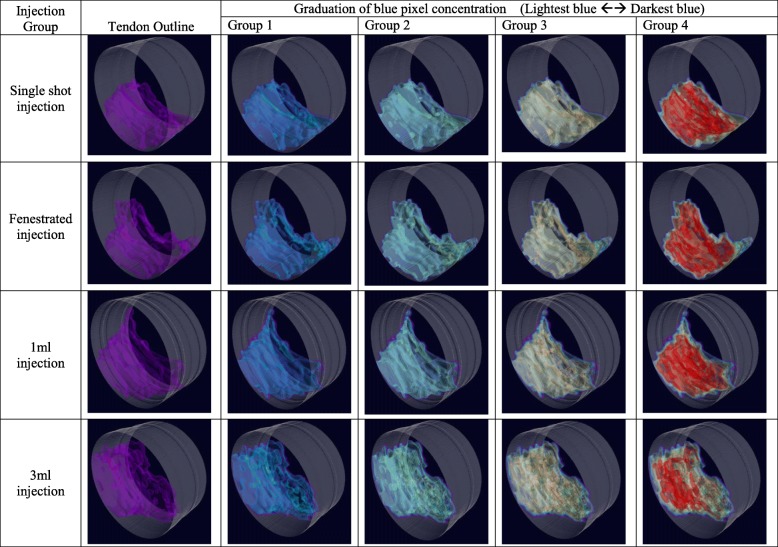
Examples of 3-Dimensional reformats of tendon segments for the four injection techniques. From left – tendon boarder, then working through four colour intensity contours. Note no pattern of pooling or longitudinal spread. (Video examples of the four groups of injections are available in the Additional file 1)

3-Dimensional reconstructions of the intratendinous injectate distributions visually confirmed broad tissue penetration centred in the midportion of the tendon with no discernable patterns of longitudinal or cross-sectional spread or pooling, or a particular anatomical localisation (e.g. to the anterior or footprint component of the tendon in the position of the ECRB) (Fig. 5).

## Discussion

This study has identified that commonly used elbow injection volumes and techniques, distribute injectate throughout 97% of the common extensor tendon in cadaveric specimens. No differences were found between injection volumes or techniques. Dye contamination of the intra-articular surface was noted in every specimen, and post-injection ultrasound revealed tears to the tendon in over 60% of specimens that were previously uninjured.

This is the first study to assess intratendinous injectate distribution of the elbow extensors in cadaveric tissue, although the use of human tissue has greater utility in its generalisability to a clinical context, the tissue morphology of cadaveric specimens should be taken into consideration. The cadaveric tissue utilised in this study was of subjects with an average age much higher (range 70–96) than the peak age of onset of LET (45-60 yrs), and though the specimens had no history of elbow surgery, their detailed medical history was not known. Pre-injection tears were noted in 60% of specimens, and though the tear rate in asymptomatic individuals is felt to be very low (Krogh et al., 2017; Ustuner et al., 2013), the proportion of tears in this study population corresponds to the tear rate seen in tendinopathic individuals, which has been reported as 57% by Walton et al. (Walton et al., 2011) and 58% by van Kollenburg et al. (van Kollenburg et al., 2009). Furthermore, calcification was noted in over half of specimens, and though the appearance of calcification is a common finding, known to increase with age, it may be of poor diagnostic value in LET (Jaén-Díaz et al., 2010). Our finding of 53% is equable to asymptomatic 50 yr. olds and is, in fact, lower than normally reported in those > 70 yrs. (Krogh et al., 2017). We, therefore, feel that the sample, though limited in number, is morphologically representative of a LET population.

Previous authors have commented upon the contamination of the elbow joint and surrounding tissues in lateral elbow injections (Keijsers et al., 2017; Park et al., 2017). However, the current literature has either assessed unguided injections in cadavers, or assessed the distribution on retrospective assessment of ultrasound images, without the known validity of such a method. Ultrasound injection is advocated in lateral elbow injections to improve accuracy and decrease contamination rate (Keijsers et al., 2017), but the present study reports that even with the application of ultrasound guidance, the joint contamination rate was 100%. The target site for elbow injection is the ECRB and though very challenging to isolate on USS from the CET, its position is deep and has a delicate and intimately associated connection to joint capsule, particularly at its anterior edge (Nimura et al., 2014). The presence of tears in this region and the propagation or creation of tears with guided injections may inevitably force the injection into the joint space.

The CET tapers from the musculotendinous junction to its origin at the lateral epicondyle. Its footprint has been defined as approximately 10 mm in anteroposterior width (Nimura et al., 2014), its true tendinous length is approximately 16 mm (Keijsers et al., 2016), its thickness at the radiocapitelar joint is 4.4 mm (Toprak et al., 2012), the average width of the CET at the musculotendinous junction though not previously defined, was 35 mm in the current study. Calculating the volume of an oblique wedge (Stocker & Harris, 1998) ($V=\frac{bh}{6\left(2a+c\right)}$*),* from these figures yields a volume of 938.66 mm^3^, which is similar to the mean total volume, derived from the pixel density, found in the current study (1040 mm^3^). The volume of 1 and 3 ml injections clearly equates to a fluid volume of 1000 and 3000 mm^3^. Therefore, with localised injection of common injectate volumes, in an area of densely packed collagen, it is unsurprising that the tendon itself is both damaged and that the injectate is disseminated down a path of least resistance. With the delicate anterior capsular edge presenting as a likely route, joint contamination becomes inevitable. Dependent on the injection substance, this finding may be clinically relevant to the practitioner, particularly in the context of a patient with an otherwise pathology free elbow joint.

Cross-sectional analysis of the CET segments, including 3-dimensional reformatting, found widespread dissemination of the injectate dye regardless of volume or technique, with no apparent pattern or pooling. Previous cadaveric and animal studies have reported a preponderance toward longitudinal spreading of injectate (Belt et al., 1993; Loftus et al., 2012; Wilson et al., 2015), in line with collagen fibre orientation. However, these studies have predominantly utilised oval tendons including lamb distal forearm extensor and flexor tendons (Wilson et al., 2015) and horse flexor tendons (Belt et al., 1993). CET tendons were included by Loftus et al. (Loftus et al., 2012) but as 14% of a cohort of predominantly hamstrings and patellar tendons, exclusively assessed with ultrasound. This is the first study to report widespread cross-sectional spread in the broad flat tendon morphology of the CET, where tears were either present or induced. Indeed, collagen cross-linking is likely to have been disrupted as part of the injection process, allowing cross-sectional spread.

The use of needle fenestration has been advocated as a method of distributing injectate more evenly (Wilson et al., 2015), however systematic review of CET injections report conflicting findings of its superiority over single shot injections (Dong et al., 2016; Fitzpatrick et al., 2017). This study did not demonstrate any statistical difference in the cross-sectional distribution of injectate between the two techniques using either 1 ml or 3 ml volumes. Further visual assessment of injectate distribution on 3D reconstruction did not isolate any noticeable difference in distribution patterns. It is important to note that significant escape of active injectate occured regardless of technique with the volumes used in this study, and the presented findings may be different if smaller volumes were employed.

This study has identified that using commonly utilised injectate volumes and techniques with ultrasound guidance into a cadaveric common extensor tendon can create tendon tears and joint contamination may be inevitable. The structural disruption to the CET seen in this study raises questions about the potentially destructive, or indeed therapeutic, effect a large injection volume may have on the CET. The disruption could be thought of as analogous to a volumetric debridement, therein an injection of 1 ml of saline may in itself have a treatment effect, and in this regard may not be an appropriate placebo intervention. Though no clinical evidence of high volume injections efficacy has been presented in LET, a 2017 randomised controlled trial of Achilles tendinopathy treatment reported them as superior to PRP injection and physiotherapy treatment [27]. Further preclinical and subsequent clinical studies of high and low volume injections are therefore warranted and future placebo randomised controlled trials may require a placebo where no fluid is injected.

### Limitations

The authors recognise several limitations present within this study. Although the number of cadaveric specimens used in the current study is greater than previously published tendon injection studies (Keijsers et al., 2017; Wiegerinck et al., 2011), the volume and technique groups are of a small number. However, variability in injectate distribution was within acceptable limits, and the statistical methods were appropriate to the small group, repeated measures design. The use of fresh frozen cadaveric material, dry arthroscopy, careful dissection practice and avoidance of freezing techniques for microtome slicing were all employed to reduce the risk of tissue destruction and degeneration not related to the injection. The choice of methylene blue was made to derive clear visualisation of contamination and to assess graduated tissue penetration. However, the dye used has a lower viscosity that some of the commonly used injectate preparations, with particular reference to PRP. Wilson et al. (Wilson et al., 2015) reported that there was no difference in the longitudinal spread of injectate in lamb tendons injected with pure methylene blue or methylene blue mixed with PRP. However, they do report cross-sectional distribution was lower in the combined group, and it, therefore, remains a possibility that that cross-sectional distribution observed in the current study may be reduced in higher viscosity injectates. The authors suggest that the volume effect, rather than viscosity, is likely to have a greater effect on collaged cross-link disruption.

## Conclusion

Both 1 ml and 3 ml injections into cadaveric elbow common extensor tendons distribute injectate equally across 97% of the intratendinous area, with no difference demonstrated between single-shot or fenestrated injection techniques. The injection of these volumes into a small anatomical space may cause damage to the tendon structure, and due to the close association of this tissue to the joint capsule, intra-articular elbow joint contamination may be inevitable, this should be taken into consideration when selecting the injection substance. In common with all cadaveric studies, the findings presented cannot be directly translated to invivo application, however, in the pursuit of optimal and justified injection technique, low volume, single shots injections are warranted clinically as broad distribution of injectate is likely and the risks of intra-articular joint contamination reduced.

## Additional file

Additional file 1: Example of a three-dimensional rendering of injectate distribution, showing no clear pattern or injection pooling or longitudinal injectate spread. (MP4 2766 kb)
